# Increased Pediatric Respiratory Syncytial Virus Case Counts Following the Emergence of SARS-CoV-2 Can Be Attributed to Changes in Testing

**DOI:** 10.1093/cid/ciae140

**Published:** 2024-04-11

**Authors:** Brittany A Petros, Carly E Milliren, Pardis C Sabeti, Al Ozonoff

**Affiliations:** Infectious Disease and Microbiome Program, Broad Institute of Massachusetts Institute of Technology and Harvard, Cambridge, Massachusetts, USA; Health Sciences & Technology Program, Harvard Medical School and Massachusetts Institute of Technology, Cambridge, Massachusetts, USA; Harvard–Massachusetts Institute of Technology MD-PhD Program, Boston, Massachusetts, USA; Department of Systems Biology, Harvard Medical School, Boston, Massachusetts, USA; Institutional Centers for Clinical and Translational Research, Boston Children's Hospital, Boston, Massachusetts, USA; Infectious Disease and Microbiome Program, Broad Institute of Massachusetts Institute of Technology and Harvard, Cambridge, Massachusetts, USA; Department of Organismic and Evolutionary Biology, Harvard University, Cambridge, Massachusetts, USA; Department of Immunology & Infectious Diseases, Harvard T. H. Chan School of Public Health, Boston, Massachusetts, USA; Howard Hughes Medical Institute, Chevy Chase, Maryland, USA; Infectious Disease and Microbiome Program, Broad Institute of Massachusetts Institute of Technology and Harvard, Cambridge, Massachusetts, USA; Precision Vaccines Program, Boston Children's Hospital, Boston, Massachusetts, USA; Department of Pediatrics, Harvard Medical School, Boston, Massachusetts, USA

**Keywords:** respiratory syncytial virus, COVID-19, viral surveillance, diagnostic testing, pediatrics

## Abstract

**Background:**

Respiratory syncytial virus (RSV) circulation dropped markedly early in the COVID-19 pandemic, followed by a resurgence with heightened case counts. The “immunity debt” hypothesis proposes that the RSV-naїve pediatric population increased during the period of low transmission. However, the evidence supporting this hypothesis is limited, and the role of changing testing practices in the perceived surge has not been comprehensively evaluated.

**Methods:**

We conducted a multicenter, retrospective analysis of 342 530 RSV encounters and 980 546 RSV diagnostic tests occurring at 32 US pediatric hospitals in 2013–2023. We used interrupted time series analysis to estimate pandemic-associated changes in RSV patient and test volume and to quantify changes in the proportions of patients requiring hospitalization, intensive care, or mechanical ventilation. We quantified the fraction of the shifts in case counts and in the age of diagnosed patients attributable to changes in testing.

**Results:**

RSV patient volume increased 2.4-fold (95% confidence interval [CI]: 1.7, 3.5) in 2021–2023 relative to the pre-pandemic phase and was accompanied by an 18.9-fold increase (95% CI: 15.0, 23.9) in RSV test volume. Shifts in patient volume and in patient age were largely attributable to increased testing. The proportions of patients with RSV that required hospitalization, intensive care, or mechanical ventilation declined significantly across all patient age groups.

**Conclusions:**

A surge in RSV testing, rather than in viral circulation, likely underlies the increased case counts observed in 2021–2023. These findings warrant a critical assessment of the immunity debt hypothesis and highlight the importance of considering the testing denominator when surveillance strategies are dynamic.

## Teaser

After the initial phase of the COVID-19 pandemic, hospitals reported an increase in RSV cases. We examined encounters at US pediatric hospitals and found that increased testing is likely driving the observed increase in case volume.

In 2021–2022 and 2022–2023, multiple countries experienced respiratory syncytial virus (RSV) epidemics with both altered seasonality and increased case counts relative to former RSV seasons [1–5]. The ongoing 2023–2024 epidemic in the United States is also notable for hospitalization rates that are unprecedented relative to years prior to the coronavirus disease 2019 (COVID-19) pandemic [6]. Viral genomic analyses have shown that multiple preexisting RSV genotypes are circulating, suggesting that viral factors are unlikely to contribute to the observed dynamics [1, 2, 4]. Instead, the prevailing hypothesis remains that alterations in human behavior (eg, social distancing and masking) during the COVID-19 pandemic reduced RSV transmission [1, 5, 7, 8], resulting in an increase in the pediatric population naïve to RSV, that is, the “immunity debt” hypothesis [9, 10].

While the immunity debt hypothesis has garnered significant attention, the evidence supporting it is limited. Multiple studies have demonstrated that pediatric RSV patients from the 2021 and 2022 seasons are older on average than in previous years [5, 7, 8, 11–13]. However, increased age has not been mechanistically tied to an immunity debt and was also observed among children diagnosed in 2020 [14], the year in which the debt was supposedly accumulating. While 2 studies have identified a decline in RSV-targeting immunoglobulin G concentrations during the pandemic [15, 16], another study found no change in neutralizing antibody titers [17] such that the clinical or epidemiological relevance of this decline is unclear. The alternative “immunity theft” hypothesis posits that COVID-19 infections weakened our immune response to other pathogens [18]. While one study found that children with an electronic health record (EHR) diagnosis of COVID-19 were more likely to receive an EHR diagnosis of RSV [19], the most likely explanation for the result is that an individual's tendency to seek healthcare for respiratory symptoms is relatively constant, regardless of the underlying etiology of illness.

The role of changing diagnostic testing practices in the large post-pandemic RSV epidemics remains to be explored. In response to COVID-19, testing capacity increased [20], and multipathogen assays that simultaneously target severe acute respiratory syndrome coronavirus 2 (SARS-CoV-2), influenza, and RSV were widely deployed [21]. Indeed, one study that reported an increase in pediatric patient age noted that RSV testing increased 5-fold [22], while another stated that all individuals admitted to the hospital for any reason were tested via multipathogen respiratory viral tests starting in 2021 [11]. Given that RSV infections with milder disease presentations, which predominantly occur in older pediatric patients, are known to escape clinical detection [13, 15], the shift in age distribution may be a consequence of the historical underestimation of the burden of RSV [23]. Increased respiratory viral testing can also address a major flaw of the immunity debt hypothesis, in that it appears not to have been repaid despite successive years of heightened case counts [18].

In this study, we aim to assess the relationship between respiratory viral testing practices and RSV epidemiology before and after the onset of the COVID-19 pandemic. We conducted a multicenter, retrospective analysis of RSV diagnostic tests and clinical encounters at 32 US pediatric hospitals between 2013 and 2023. We quantified changes in test volume and assessed the degree to which perceived shifts in the epidemiology of RSV can be attributed to altered testing practices.

## METHODS

### Data Acquisition and Ethics Statement

We extracted data from the Pediatric Health Information System (PHIS) [24], which contains administrative data from a network of 48 US tertiary care children's hospitals that span all 9 geographic census divisions. We isolated emergency department (ED) visits and hospitalizations associated with an RSV diagnosis, an RSV diagnostic test, an influenza virus diagnosis, or an influenza diagnostic test provided that the encounter discharge date was between 1 July 2013 and 30 June 2023, the associated hospital submitted data every month of the study period from both ED and inpatient units (32 of 48 hospitals), and the patient was aged <18 years (Supplementary Methods).

Because we used deidentified data, this study was considered nonhuman subjects research, exempt from requiring institutional review board (IRB) approval, per the policies of Boston Children's Hospital IRB and per an exempt determination at the Broad Institute.

### Statistical Analyses

Analyses were conducted in R v.4.1.1, with temporal data analyzed at the resolution of month of discharge date. Demographic and clinical characteristics of patients were compared between the pre-COVID-19-pandemic (“pre-pandemic”) and post-SARS-CoV-2-emergence (“post-emergence”) phases. The pre-pandemic phase was defined as ending on 1 April 2020, the date at which more than 70% of states had issued stay-at-home orders [25]. The post-emergence phase was defined as starting on the first month in which RSV patient volume exceeded that of April 2020, signifying the resumption of annual RSV epidemics. Binary variables were compared using Fisher's exact test; continuous variables were compared using the Wilcoxon rank sum test; and categorical variables were compared using *χ*^2^ tests. Two-sample *z* tests were used to test for differences in the proportions of patients within an age stratum that required hospitalization, intensive care, or mechanical ventilation across the 2 phases.

We conducted interrupted time series (ITS) analysis to identify trends in encounter and testing volume, considering both linear and log-linear models with the following independent variables: time, indicator variables for the intermediate period (*I_pand_*) and the post-emergence phase (*I_PE_*), variables that enabled a change in slope for each phase, and harmonic terms to model seasonality (*H_0_*, *H_pand_*, and *H_PE_*). We constructed models with all combinations of 0–2 harmonic terms per phase (ie, seasonality was independently modeled for each phase using maximally 1 sine and 1 cosine term), selecting the model that minimized the transformation-adjusted Akaike information criterion [26]. We also conducted ITS analysis to estimate the effects of pandemic-related restrictions on the proportions of RSV tests that were positive, ED patients admitted to the hospital, inpatients who required intensive care, and inpatients who received mechanical ventilation, considering linear models with up to 4 harmonic terms per phase (ie, seasonality was independently modeled for each phase using maximally 2 sine and 2 cosine terms). For log-linear models, the equations were as follows:


$$\begin{array}{rl} log(volume)\;= & \;\alpha_{0}\;+\;\alpha_{1}I_{\;pand}\;+\;\alpha_{2}I_{PE}\;+\;\beta_{0\;}t+\;\beta_{1}I_{\;pand}\;t\\ & +\beta_{2}I_{PE}t+H_{0}\;+H_{\;pand}+H_{PE} \end{array}$$

allowing us to calculate the fold change in the post-emergence phase, relative to the pre-pandemic phase, as follows:


$$fold\;change=\;exp(\alpha_{2})$$

For linear models, the equations were as follows:


$$\begin{array}{rl} proportion\;= & \;\alpha_{0}+\alpha_{1}I_{\;pand}+\alpha_{2}I_{PE}+\beta_{0\;}t+\beta_{1}I_{\;pand}\;t\\ & +\beta_{2}I_{PE}t+H_{0}+H_{\;pand}+H_{PE} \end{array}$$

such that the additive change in the post-emergence phase, relative to the pre-pandemic phase, was $\;\alpha_{2}$.

We conducted 2 modified bootstrap analyses (ie, 50 replicates of resampling with replacement) to estimate the effects of testing changes on RSV patient volume and RSV patient age. First, we estimated post-emergence test volumes under the counterfactual (ie, no pandemic-associated disruptions) by considering linear and log-linear models of pre-pandemic test volumes as a function of time. We then bootstrapped post-emergence tests according to the predicted monthly test volumes. We tallied the counterfactual monthly patient volume by summing the number of patients who received a clinical diagnosis of RSV and the number of positive post-emergence tests in the bootstrap sample. Second, we bootstrapped the post-emergence testing data according to the age distribution (in 90-day bins) derived from the pre-pandemic testing data. The counterfactual patient age distribution consisted of the ages of patients whose positive tests were present in the bootstrap sample.

## RESULTS

### Increases in RSV Patient Volume Can Be Attributed to Changing Testing Paradigms

Between July 2013 and June 2023, across 32 geographically diverse pediatric hospitals, 980 546 RSV diagnostic tests were conducted and 342 530 clinical encounters associated with an RSV diagnosis occurred. We divided the study period into 2 phases by analyzing monthly patient volume: the pre-pandemic phase (July 2013–March 2020) and the post-emergence phase (April 2021–July 2023). No RSV epidemic occurred in the intermediate pandemic year, with a median of 46.5 monthly patients (interquartile range [IQR], 32–78.5) diagnosed with RSV across all 32 hospitals; this period was thus excluded from further study.

**Figure 1.**
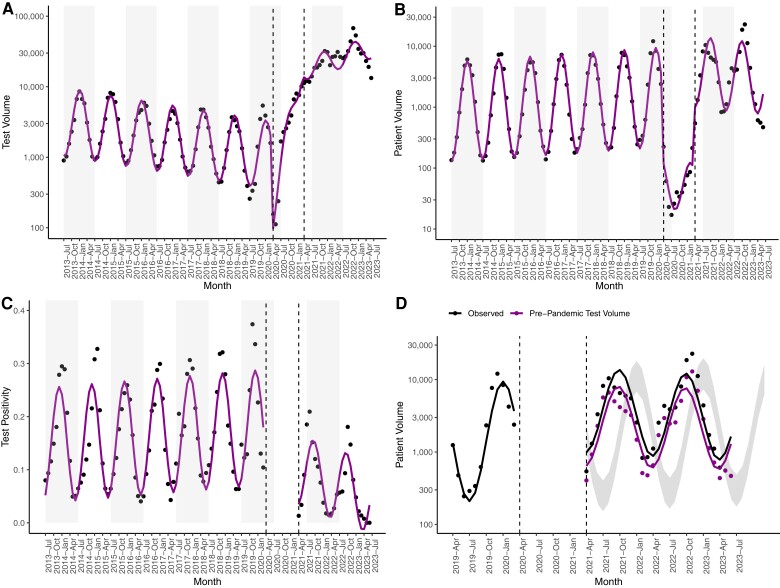
Monthly respiratory syncytial virus test volume (*A*), patient volume (*B*), and test positivity (*C*). Black dots signify observed values, with model fits in magenta. Gray rectangles denote alternating years (from July–July). *D*, Monthly patient volume from April 2019 to July 2023. Observed patient volume is compared to simulated patient volume under test volumes predicted via pre-pandemic testing data. Dots signify observed values, with model fits as lines. The prediction interval depicts patient volumes predicted from pre-pandemic patient volumes. *A–D*, Dashed lines delineate the end of the pre-pandemic phase and the start of the post-emergence phase.

First, we examined temporal trends in RSV test and patient volume via ITS analysis. We quantified deviations in volume that existed above and beyond the secular trends (eg, an annual increase in RSV patient volume observed over multiple years). RSV test volume declined in the pre-pandemic phase, then demonstrated a remarkable increase by a factor of 18.9 (95% confidence interval [CI]: 15.0, 23.9) in the post-emergence phase (Figure 1*A*, Supplementary Table 1). The bulk of the additional RSV testing was conducted via molecular tests that simultaneously detect SARS-CoV-2 and other respiratory pathogens (“SARS-CoV-2 multipathogen tests”; Supplementary Figure 1), which made up 89.8% of tests conducted in the post-emergence period. The volume of patients diagnosed with RSV increased by a factor of 2.4 (95% CI: 1.7, 3.5) in the post-emergence phase (Figure 1*B*, Supplementary Table 1). While case detection increased, test positivity dropped significantly from 14.5% (95% CI: 12.8%, 16.3%) in the pre-pandemic phase to 6.1% (95% CI: 1.9%, 10.3%) in the post-emergence phase (Figure 1*C*, Supplementary Table 1). Though the seasonality of RSV changed from the pre-pandemic phase to the post-emergence phase (Figure 1*D*), post-emergence patient volumes fell within the upper and lower boundaries of the 95% prediction interval derived from pre-pandemic data for 26 of 27 (96%) months.

Though the SARS-CoV-2 multipathogen tests can also diagnose influenza virus, influenza patient volume did not increase in the post-emergence phase (Supplementary Figure 2*A*, Supplementary Table 1). While RSV test volumes increased by a factor of 18.9 (95% CI: 15.0, 23.9) in the post-emergence phase, influenza test volume increased by a factor of 3.0 (95% CI: 2.0, 4.3; Supplementary Table 1). In other words, influenza testing was more robust than RSV testing prior to the emergence of SARS-CoV-2, with the introduction of SARS-CoV-2 multipathogen tests resulting in a smaller increase in influenza test volume (Supplementary Figure 2*B*).

To assess the impact of increased testing on increased RSV patient volume, we simulated the counterfactual scenario in which post-emergence test volume was consistent with pre-pandemic test volume (see the Methods section). Under this scenario, RSV patient volume increased by a factor of 1.37 (95% CI: .96, 1.95; not significant) in the post-emergence phase (Figure 1*D*, Supplementary Table 1) such that 74.2% of the observed increase in patient volume can be attributed to increased testing. Across all 50 simulations, the fold change in RSV patient volume was not significant, implying that increased testing was necessary for detection of an increase in patient volume. In summary, both RSV test and patient volume increased in the post-emergence phase, with changes in patient volume largely attributable to changes to testing.

### Increased Testing of Older Children Is Associated With Increased Patient Age

**Figure 2.**
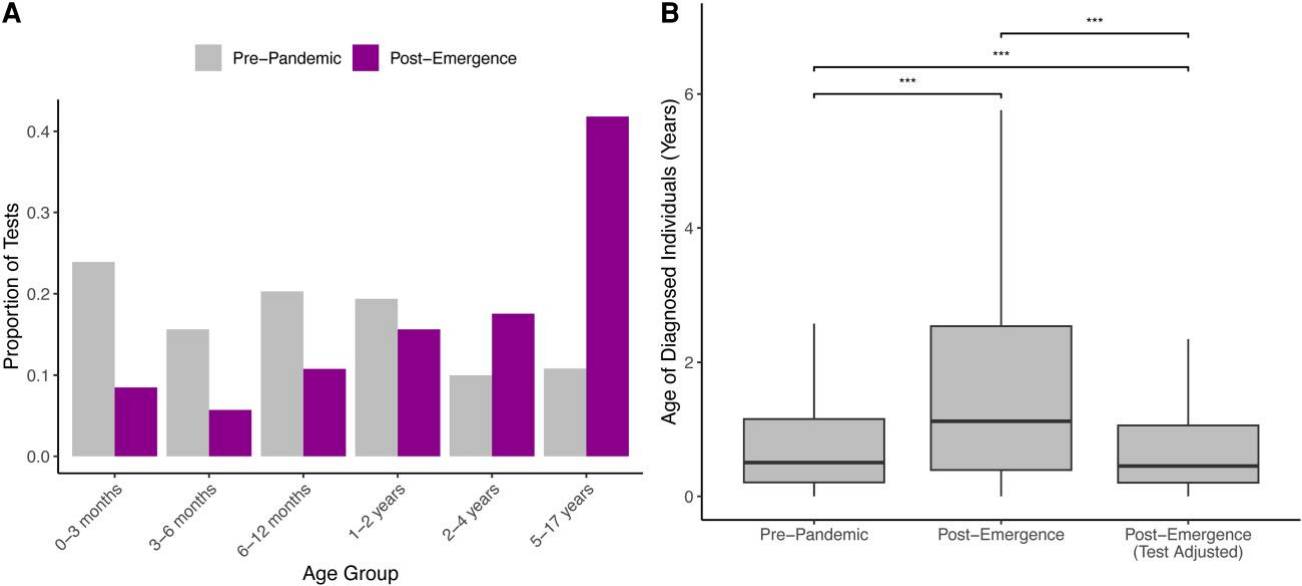
*A*, Proportion of respiratory syncytial virus (RSV) tests conducted on each age group in the pre-pandemic vs post-emergence phase. *B*, Age, in years, of patients tested for and diagnosed with RSV in the pre-pandemic and post-emergence phases. Because tests in the post-emergence phase were conducted on older patients, on average, than in the pre-pandemic phase, the post-emergence testing data were bootstrapped according to the age distribution of the pre-pandemic testing data. The resulting patient age distribution is reported as “Post-Emergence (Test Adjusted).” Box plots display the first, second, and third quartiles, with whiskers extending to the minimum of 1.5 times the interquartile range and the most extreme data point in either direction. ***, *P* < .001 via Wilcoxon rank sum test.

Next, we compared the demographic and clinical characteristics of patients tested for and diagnosed with RSV in the pre-pandemic and post-emergence phases. Patients tested for RSV differed across the phases among multiple demographic axes (Table 1), the most striking of which was age, which increased from a median of 8.6 months to 35.1 months (*P* < .001; Table 1, Figure 2*A*, Supplementary Figure 3). Patients tested for RSV were also significantly less likely to have a complex chronic condition (CCC) [27] in the post-emergence phase, defined as a medical condition expected to last at least 12 months that requires specialty care and periods of hospitalization at a tertiary care center (15.8% vs 18.5%, *P* < .001; Table 1) [28]. A larger proportion of RSV tests was conducted on older and healthier children in the post-emergence phase, and test positivity dropped significantly across all age groups (*P* < .001; Table 1).

**Table 1.** Demographic Characteristics of Patients Tested for Respiratory Syncytial Virus

Characteristic	Pre-Pandemic Phase July 2013–March 2020 N = 210 424 Tests	Post-Emergence Phase April 2021–June 2023 N = 723 460 Tests	*P* Value
Age, m	8.57 (3.12–20.01)	35.05 (11.99–88.21)	<.001
Age category, %			
0–90 d 3–6 m 6–12 m 1–2 y 2–4 y 5–17 y	23.91 15.63 20.29 19.38 9.99 10.81	8.50 5.73 10.77 15.63 17.57 41.80	<.001
Sex, % male	55.84	54.19	<.001
Race, %			
White Black Asian Pacific Islander American Indian Other Multiple Unknown	43.94 24.00 2.31 0.35 0.23 9.91 1.15 18.11	48.89 23.07 4.33 0.45 0.35 13.73 3.22 5.96	<.001
Ethnicity, %			
Hispanic or Latino Not Hispanic or Latino Unknown	31.59 62.80 5.61	30.67 63.81 5.51	<.001
Complex chronic condition by age group, %	18.45	15.84	<.001
0–90 d 3–6 m 6–12 m 1–2 y 2–4 y 5+ y	13.77 10.55 11.39 14.64 27.68 51.75	14.58 17.38 11.62 10.02 8.50 2.23	<.001 <.001 <.001 <.001 <.001 <.001
Premature or neonatal complex chronic condition, %	2.71	1.64	<.001
Patient type			
ED (discharge) ED and IP (ED admit) IP (direct admit)	56.60 34.73 8.67	66.34 27.00 6.66	<.001
Diagnostic test, %			
Culture Antigen PCR DNA Probe SARS-CoV-2 multipathogen Other/Unspecified test Multiple tests	3.05 83.39 5.26 4.77 0 0.01 3.52	0.26 7.52 10.78 0.14 80.78 <0.01 0.52	<.001
Test positivity by diagnostic, %	21.50%	7.48%	<.001
Culture Antigen PCR DNA probe SARS-CoV-2 multipathogen	9.64 23.03 18.16 7.68 NA	9.24 12.02 10.97 6.20 6.59	.615 <.001 <.001 .090 NA
Test positivity by age group, %			
0–90 d 3–6 m 6–12 m 1–2 y 2–4 y 5+ years	26.30 27.47 22.62 20.35 18.03 5.46	14.58 17.38 11.62 10.02 8.50 2.23	<.001 <.001 <.001 <.001 <.001 <.001

Medians (with interquartile ranges) were used to summarize distributions of continuous variables.

Abbreviations: ED, emergency department; IP, inpatient; NA, not applicable; PCR, polymerase chain reaction; SARS-CoV-2, severe acute respiratory syndrome coronavirus 2.

Among individuals diagnosed with RSV, patient age also increased from a median of 6.9 months to 11.9 months (*P* < .001; Table 2, Figure 2*B*). Patients diagnosed with RSV were significantly less likely to have a CCC in the post-emergence phase (11.7% vs 16.8%, *P* < .001; Table 2). Moreover, the apparent clinical severity of RSV cases declined in the post-emergence phase, with patients less likely to be admitted from the ED (38.7% vs 50.6%); to require intensive care (27.2% vs 32.3%), mechanical ventilation (7.4% vs 11.9%), or extracorporeal membrane oxygenation (0.15% vs 0.22%); or to expire in the hospital (0.16% vs 0.24%; Table 2).

**Table 2.** Demographic and Clinical Characteristics of Patients Diagnosed With Respiratory Syncytial Virus

Characteristic	Pre-Pandemic Phase July 2013–March 2020 N = 202 131 Encounters	Post-Emergence Phase April 2021–June 2023 N = 139 506 Encounters	*P* Value
Demographic characteristics			
Age, m	6.87 (2.60–17.48)	11.89 (4.20–28.32)	<.001
Age category, %			
0–90 d 3–6 m 6–12 m 1–2 y 2–4 y 5+ y	27.99 17.85 18.85 17.72 11.16 6.43	18.18 14.56 17.40 20.00 18.46 11.40	<.001
Sex, % male	55.31	54.75	.001
Race, %			
White Black Asian Pacific Islander American Indian Other Multiple Unknown	58.61 18.43 2.80 0.53 0.29 10.31 1.49 7.54	57.03 19.15 3.30 0.80 0.30 11.21 2.72 5.49	<.001
Ethnicity, %			
Hispanic or Latino Not Hispanic or Latino Unknown	25.45 69.83 4.72	27.09 67.74 5.17	<.001
Complex chronic condition by age group, %	16.84	11.69	<.001
0–90 d 3–6 m 6–12 m 1–2 y 2–4 y 5 + y	12.14 8.91 11.89 16.67 29.22 52.76	11.98 6.46 7.74 9.45 13.21 25.46	.50 <.001 <.001 <.001 <.001 <.001
Premature or neonatal complex chronic condition, %	3.10	2.57	<.001
Patient type, %			
ED (discharge) ED and IP (ED admit) IP (direct admit)	31.13 52.38 16.49	44.58 46.39 9.03	<.001
Diagnostic test, %			
Culture Antigen Polymerase chain reaction DNA probe Severe acute respiratory syndrome coronavirus 2 multipathogen Other/Unspecified test Multiple tests None (clinical diagnosis)	0.28 20.29 1.09 0.27 0 <0.01 0.44 77.62	0.13 4.65 2.63 0.04 31.04 <0.01 0.29 61.22	<.001
Clinical characteristics			
ED patients hospitalized, %	50.55	38.70	<.001
IP length of stay, d	3 (2–5)	2 (1–4)	<.001
IP length of stay category, %			
0–1 d 1–2 d 2–4 d 5–7 d >7 d	3.10 19.91 36.62 22.90 17.47	3.76 23.35 39.83 20.99 12.08	<.001
IPs admitted to the intensive care unit or neonatal intensive care unit, %	32.25	27.16	<.001
IPs mechanically ventilated, %	11.90	7.36	<.001
IPs receiving extracorporeal membrane oxygenation, %	0.22	0.15	<.001
IP hospital mortality, %	0.24	0.16	<.001
Concurrent influenza diagnosis, %	2.19	1.38	<.001
Concurrent coronavirus disease 2019 diagnosis, %	0	2.73	<.001

Medians (with interquartile ranges) were used to summarize distributions of continuous variables.

Abbreviations: ED, emergency department; IP, inpatient.

Among pediatric patients, the youngest patients [29–31] are at the greatest risk for severe disease; therefore, true shifts in patient age could meaningfully impact RSV morbidity. However, we hypothesized that the apparent shifts were attributable to shifts in the age of those tested for RSV (Figure 2*A*, Supplementary Figure 3). We bootstrapped post-emergence tests according to the age distribution of the pre-pandemic testing data (see the Methods section) to assess the impact of increased testing of older children on the age of children diagnosed with RSV. Under this scenario in which the age of patients tested for RSV was held constant across phases (Supplementary Figure 3) and a diagnostic test was a prerequisite to RSV diagnosis, the age of patients diagnosed with RSV exhibited a statistically significant, albeit clinically insignificant, decline, from a median of 6.0 months (IQR, 2.5–13.9) in the pre-pandemic phase to 5.4 months (IQR, 2.5–12.7) in the post-emergence phase (*P* < .001; Figure 2*B*). This result, observed across all 50 bootstrap replicates, suggests that the apparent increase in patient age following the emergence of SARS-CoV-2 can be ascribed to changes in the age of patients tested for RSV.

### The Apparent Declines in Clinical Severity Are Specific to RSV

**Figure 3.**
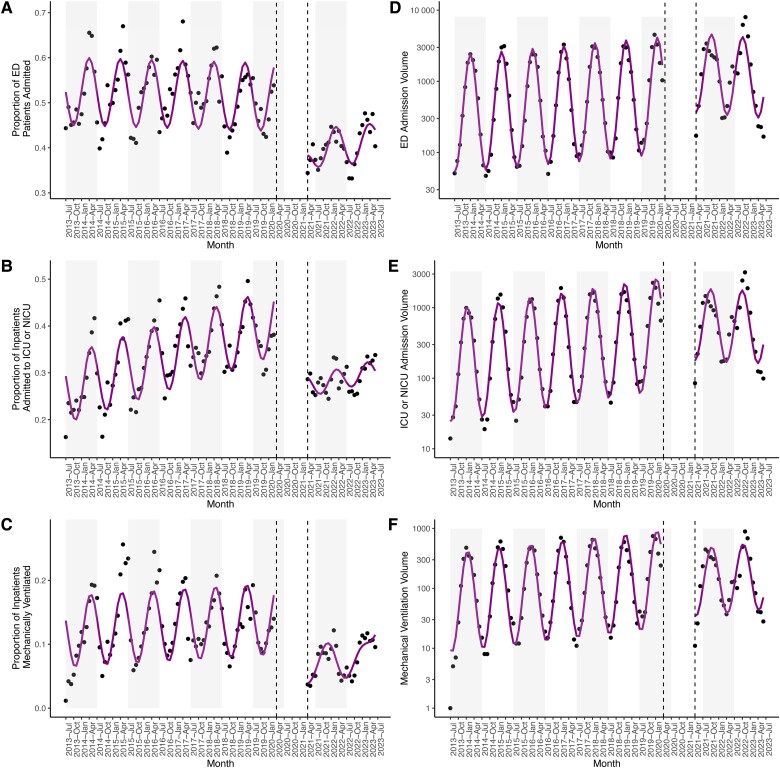
Proportion (*A*) and volume (*D*) of patients with a diagnosis of respiratory syncytial virus (RSV) admitted from the ED. Proportion (*B*) and volume (*E*) of inpatients with a diagnosis of RSV admitted to the ICU or NICU. Proportion (*C*) and volume (*F*) of inpatients with a diagnosis of RSV mechanically ventilated. *A–F*, Black dots signify observed values, with model fits in magenta. Gray rectangles denote alternating years (from July to July). Dashed lines delineate the end of the pre-pandemic phase and the start of the post-emergence phase. Data in the interim period are not shown. Abbreviations: ED, emergency department; ICU, intensive care unit; NICU, neonatal intensive care unit.

Declining measures of clinical severity were observed among RSV cases (Table 2) and may reflect increased detection of cases with milder disease presentations or decreased availability of healthcare resources in the context of the COVID-19 pandemic. We therefore examined temporal trends in clinical severity for patients with RSV and for patients with influenza virus. The fraction of patients admitted from the ED dropped from 52.6% (95% CI: 50.6%, 54.6%) in the pre-pandemic phase to 37.7% (95% CI: 32.6%, 42.9%) in the post-emergence phase for RSV (Figure 3*A*, Supplementary Table 2). In contrast, the proportion of patients diagnosed with influenza virus who were admitted from the ED increased in the post-emergence phase (Supplementary Figure 4*A*, Supplementary Table 2). The proportion of inpatients admitted to an intensive care unit (ICU) also declined among those diagnosed with RSV from 26.3% (95% CI: 24.5%, 28.2%) to 10.4% (95% CI: 6.1%, 14.7%; Figure 3*B*, Supplementary Table 2) but remained stable for patients with influenza (Supplementary Figure 4*B*, Supplementary Table 2). Moreover, the fraction of patients with RSV who received mechanical ventilation declined by 10.3% (95% CI: −14.1%, −6.5%; Figure 3*C*, Supplementary Table 2), though there was no change among those diagnosed with influenza (Supplementary Figure 4*C*, Supplementary Table 2). In summary, patients diagnosed with RSV, but not influenza virus, displayed milder disease presentations in the post-emergence phase.

### The Volume of Severe RSV Cases Remains Stable

We also analyzed the volume of RSV patients that demonstrated severe disease, as elevated patient volumes have the potential to overwhelm healthcare systems. Both overall RSV patient volume and the volume of patients hospitalized from the ED increased in the post-emergence phase by 2.4-fold (95% CI: 1.7, 3.5; Figure 1*B*) and 1.9-fold (95% CI: 1.3, 2.9; Figure 3*D*, Supplementary Table 1), respectively. However, the volume of patients with RSV who required intensive care (95% CI: .80, 1.7) or mechanical ventilation (95% CI: .49, 1.2) remained stable in the post-emergence phase relative to the pre-pandemic phase (Figure 3*E* and *F*, Supplementary Table 1). While significantly more RSV cases were detected in the post-emergence phase, there is no evidence for an increase in the number of severe cases.

### Apparent Declines in RSV Clinical Severity Are Present Across Patient Age Strata

**Figure 4.**
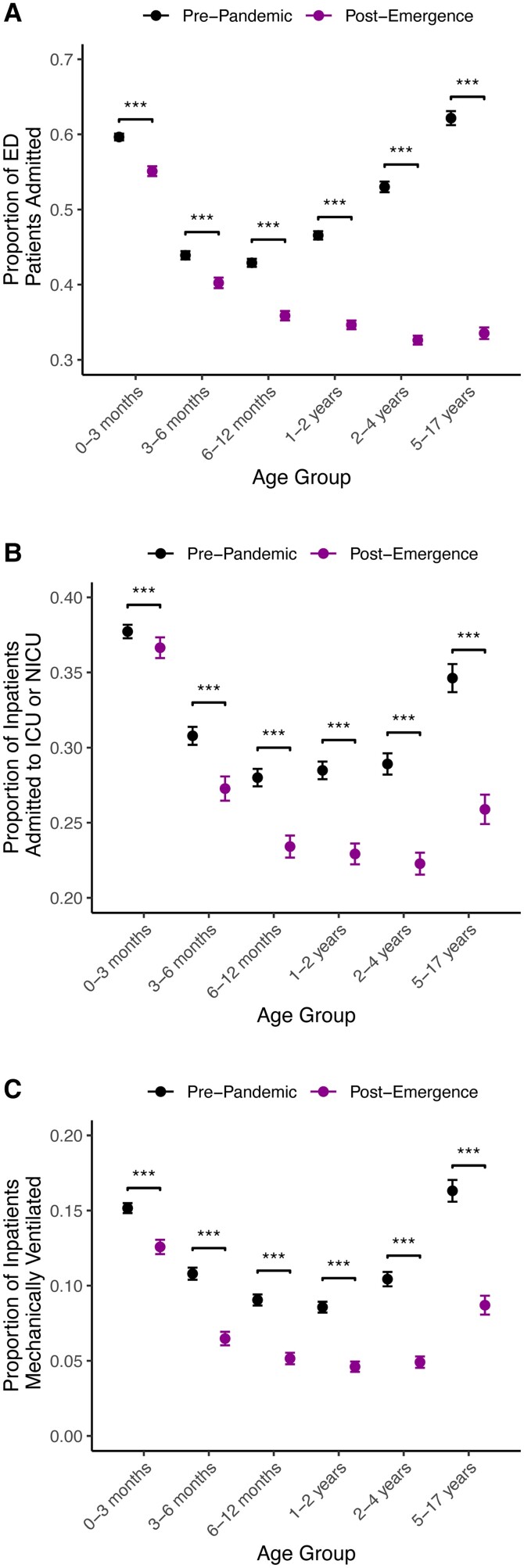
Proportions of ED patients admitted to the hospital (*A*), inpatients admitted to the ICU or NICU (*B*), or inpatients who received mechanical ventilation (*C*) for patients with a diagnosis of RSV by age group and phase. *, *P* < .05; **, *P* < .01; ***, *P* < .001. Abbreviations: ED, emergency department; ICU, intensive care unit; NICU, neonatal intensive care unit; RSV, respiratory syncytial virus.

Given known relationships between RSV severity and pediatric patient age [29–31], it is possible the declining measures of clinical severity could be attributed solely to the observed increase in patient age. However, we observed significant declines in the proportion of patients who were admitted from the ED, the proportion of patients admitted to the ICU or neonatal ICU, and the proportion of patients receiving mechanical ventilation within every age group (Figure 4). Among patients with RSV evaluated in the ED, the percent change in the admission proportion declined monotonically with patient age from 7.6% (0–3 months) to 46.1% (5–17 years; Figure 4*A*, Supplementary Table 3). Similarly, the percent change in the proportion of inpatients admitted to the ICU decreased monotonically with patient age from 2.9% (0–3 months) to 25.2% (5–17 years; Figure 4*B*, Supplementary Table 3), and the greatest declines in the proportion of inpatients mechanically ventilated occurred among children aged >6 months (Figure 4*C*, Supplementary Table 3). Though present across age groups, reductions in clinical severity were most pronounced among older patients, reflecting the pre-pandemic practice of primarily testing older children who exhibited severe disease.

## DISCUSSION

Using administrative data from 32 pediatric hospitals in the United States, we quantified changes in the epidemiology of RSV over the last decade. While RSV patient volume doubled after the lifting of COVID-19 pandemic mitigation measures, we found that RSV test volume increased over 18-fold. We identified an increase in patient age that can be explained by the increased testing of older children. We also documented declines in measures of clinical acuity, including the proportions of patients who required hospitalization, intensive care, or mechanical ventilation, across all patient age groups, including in patients aged <12 months.

**Figure 5.**
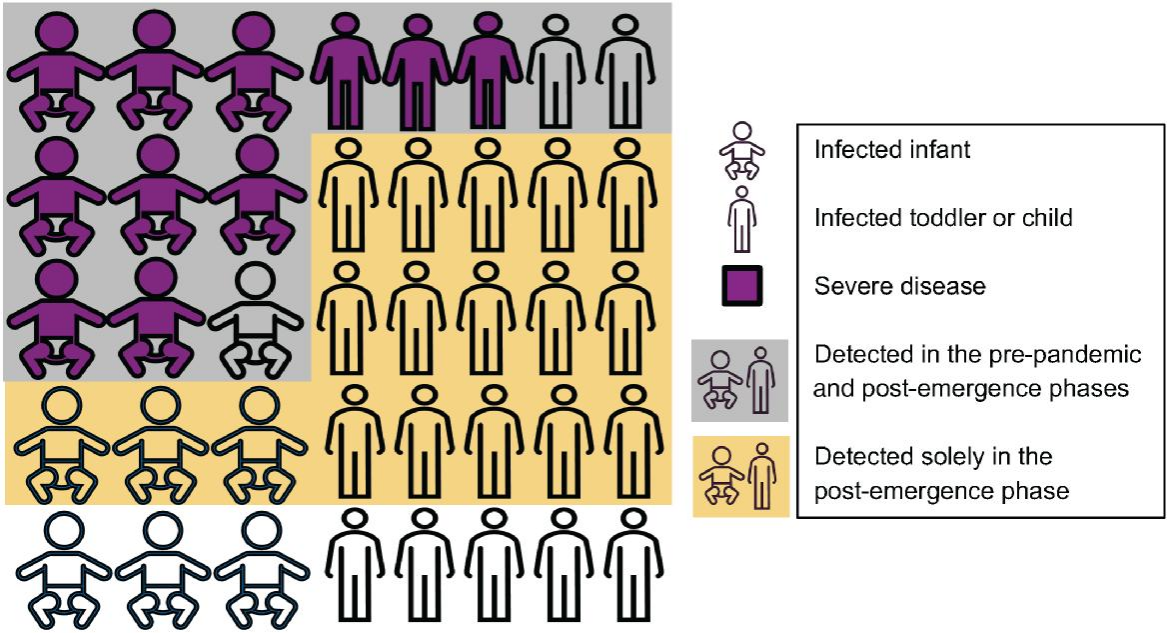
Schematic depiction of the study's conclusions. The most severe cases of respiratory syncytial virus (RSV), depicted in magenta, were detected in the pre-pandemic phase and continued to be detected in the post-emergence phase. Thus, there was no increase in the volume of patients who required intensive care or mechanical ventilation. However, additional testing in the post-emergence phase resulted in the additional detection of primarily mild or moderate RSV cases, leading to greater patient volume. The average age of diagnosed individuals increased, and the fraction of patients who experienced clinically severe outcomes decreased, as infections in older children, who are at lower risk for severe disease, were less likely to be detected in the pre-pandemic phase.

Increased patient volume and an apparent decline in clinical severity are expected consequences of large increases in RSV testing. Patients with the most severe clinical courses rarely escape detection, with increased testing predominantly capturing those with milder illness and decreasing the apparent severity of infection regardless of age (Figure 5). Because the risk of severe RSV declines with increased pediatric patient age [29–31], the detection of more mild cases shifts the average patient age upward. We found that the proportion of clinically severe cases was remarkably high among children aged 5–17 years in the pre-pandemic phase (Figure 4), reflecting underascertainment of milder RSV infections prior to the emergence of COVID-19 [23]. Moreover, we identified increased detection of RSV in between annual epidemics in the post-emergence phase (Figure 1*B*), likely due to heightened RSV testing regardless of the season (Figure 1*A*).

We considered multiple other possible explanations for our results. While it remains possible that RSV circulation simultaneously increased due to decreased population-level immunity, this hypothesis is difficult to reconcile with the finding that the volumes of patients who required intensive care or mechanical ventilation were unchanged. Indeed, the clinical significance of the immunity debt paradigm remains unknown and may be difficult to quantify given the concurrent surge in RSV testing. The observed decline in RSV clinical severity is also unlikely to be the result of hospital capacity limitations, given that no equivalent decline was noted for patients with influenza virus. Additionally, while the increase in patient age may also be impacted by a pandemic-associated increase in the tendency to seek healthcare for respiratory symptoms, testing alone can explain the increase.

There are limitations to our work. Our study was observational, and the associations that we identified may or may not be causal. PHIS contains data on tertiary care pediatric hospitals, which may not generalize to all hospitals that provided care for pediatric patients with RSV. We defined our patient cohorts using *International Classification of Diseases, Ninth Revision, Clinical Modification*, and *International Classification of Diseases, Tenth Revision, Clinical Modification*, codes present in billing data, which may underestimate or overestimate the true patient volumes. Furthermore, we cannot discern ED visits and admissions specifically for RSV-related symptoms, and it is possible that some of the identified cases were incidental.

Nevertheless, our work has an important takeaway that seems to have been unaddressed in recent literature regarding RSV activity in the United States. Endemic pathogen surveillance is complex, and case surges are often contemporaneous with testing surges [32]. It thus becomes essential that we consider the shifting testing denominator when comparing metrics such as case counts to those collected in the past. As testing becomes more widespread such that an increased fraction of the true infections is detected, we move closer to the ground truth in terms of case severity and fatality rates. However, increased testing is not universally beneficial, as high-volume testing can deflate the positive predictive value during periods of low viral circulation. Approaches such as wastewater-based epidemiology [33, 34] can mitigate the biases induced by changes in testing and will be important data sources for improved surveillance of RSV and other respiratory pathogens.

## Supplementary Data

Supplementary materials are available at *Clinical Infectious Diseases* online. Consisting of data provided by the authors to benefit the reader, the posted materials are not copyedited and are the sole responsibility of the authors, so questions or comments should be addressed to the corresponding author.

## Supplementary Material

ciae140_Supplementary_Data

## Notes

***Acknowledgments.*** Roby Bhattacharyya, Bronwyn MacInnis, and Gage Moreno provided thoughtful feedback on this work.

***Financial support***. This work was supported by the National Institute of General Medical Sciences (T32GM00773 and T32GM144273 to B. A. P.), the MD-PhD Broad Fellowship (to B.A.P.), the Howard Hughes Medical Institute Investigator Program (to P. C. S.), and the National Institute of Allergy and Infectious Diseases (U19AI110818 to P. C. S.).

***Data sharing.*** Data analyzed in this study were downloaded from the Pediatric Health Information System database (https://www.childrenshospitals.org/content/topics/data-insights), which prohibits data sharing outside of its member hospitals. Scripts used to analyze the data are available at https://github.com/bpetros95/rsv-testing.
